# CAR-T cell exhaustion in B cell lymphoma: Current status, mechanisms, and potential solutions

**DOI:** 10.1016/j.omton.2025.201099

**Published:** 2025-11-24

**Authors:** Ruocong Zhao, Jianglong Xia, Yuanyuan Zheng, Wenfa Huang, Li Yu, Lixin Wang

**Affiliations:** 1 Department of Hematology and Oncology, Shenzhen University General Hospital, Shenzhen, China; 2 International Cancer Center, Shenzhen Key Laboratory, Hematology Institution of Shenzhen University, Shenzhen University Health Science Center, Shenzhen University, Shenzhen, China

**Keywords:** MT: Regular Issue, chimeric antigen receptor, lymphoma, exhaustion, cell therapy

## Abstract

Chimeric antigen receptor T cell (CAR-T) therapy represents a revolutionary approach in the treatment of refractory or relapsed hematological malignancies including lymphoma. Despite its efficacy, a significant subset of patients experiences disease progression or relapse after initial response, and CAR-T cell exhaustion in the tumor microenvironment (TME) is a critical cause for the unsatisfactory responses. This review discusses the current situation of CAR-T therapies in the clinical treatment of lymphoma patients, the mechanisms of CAR-T cell exhaustion, and potential strategies to overcome CAR-T cell exhaustion, thereby bringing hope to improve therapeutic outcomes for lymphoma patients.

## Graphical abstract

**Figure:**
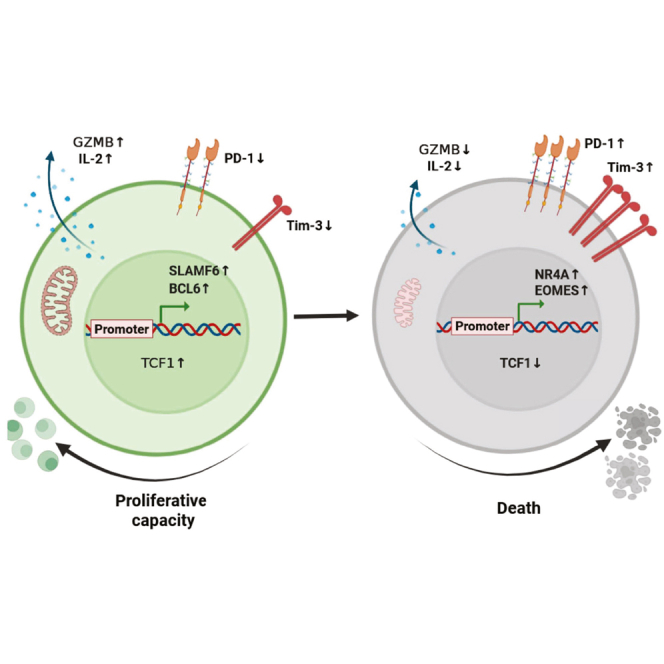

## Teaser

Zhao and colleagues reviewed the current situation of CAR-T therapies in the clinical treatment of lymphoma patients, the mechanisms of CAR-T cell exhaustion, and potential strategies to overcome CAR-T cell exhaustion for lymphoma patients.

## Introduction

Immunotherapies including immune checkpoint inhibitors and adoptive cell transfer have revolutionized the clinical treatment of cancer and these advances have led to substantial research interest in T cell exhaustion. T cell exhaustion results from persistent exposure to antigen, characterized by the loss of T cell effector function and proliferation capacity and high expression of multiple inhibitory receptors including PD-1, TIM-3, and LAG-3.^1^^,^^2^^,^^3^ It has been accepted that chronic T cell receptor (TCR) signaling is a core mechanistic driver of exhaustion and is highlighted by the well-established role of TCR-responsive transcription factors that maintain the exhausted states of T cells.^4^^,^^5^^,^^6^ Besides, many studies have revealed other factors such as inflammatory microenvironment and aberrant metabolism in the tumor microenvironment (TME) that also contribute to functional exhaustion of T cells. Given the reported mechanisms, targeting strategies are being actively developed to enhance the efficacy of current immunotherapies or to bring benefit to non-responders.

In recent years, CAR-T cells have shown promising efficacy and become the second line of standard treatment for relapsed/refractory B cell lymphoma; however, the complete remission (CR) rate of CAR-T therapy in lymphoma is not as high as in lymphoblastic leukemia, and a majority of patients will relapse within 2 years.^7^^,^^8^^,^^9^ Of note, suppressive TME in lymphoma has been accepted to negatively affect CAR-T cell activity and induces CAR-T cell exhaustion to abolish persistent antitumor activity.^10^^,^^11^^,^^12^^,^^13^ In this review, we first summarize the clinical data for CAR-T cell therapy in the treatment of lymphoma patients, then we present the recent advances in understanding CAR-T cell exhaustion. Finally, we summarize those potential strategies to overcome CAR-T cell exhaustion. Collectively, we provide a detailed overview for CAR-T cell exhaustion from both pre-clinical and clinical research under the background of lymphoma.

## Clinical status of CAR-T therapy in lymphoma

According to the World Health Organization, the annual growth rate of lymphoma incidence is between 5% and 7%, with more than 200,000 deaths a year.^14^^,^^15^ Lymphoma is a heterogeneous group of diseases including Hodgkin lymphoma (HL) and non-Hodgkin lymphoma (NHL). NHL is divided into two categories: aggressive (such as diffuse large B cell lymphoma [DLBCL]) and inert (follicular and marginal zone lymphoma).^16^^,^^17^ For aggressive lymphoma, 40%–50% of patients are clinically cured by immunochemotherapy, while the other patients are relapsed or refractory (R/R) lymphoma, who have extremely poor prognosis if not suitable for hematopoietic stem cell transplantation (HSCT) therapy or relapse after transplantation.^15^^,^^18^^,^^19^ Intriguingly, remarkable results were observed in clinical trials using CAR-T cell therapy to treat R/R lymphoma. Axicabtagene Ciloleucel (axi-cel) is CD19 targeted CAR-T cell therapy developed by Kite Pharma. Results of the 2019 ZUMA-1 study showed that in 111 patients with relapsed/refractory large B cell lymphoma (R/R LBCL) treated with axi-cel for third-line patients, the overall response rate (ORR) and CR rate were 82% and 54%. At 5-year follow-up, 31% of patients achieved a sustained response, and the estimated 5-year overall survival (OS) rate was 42%, suggesting that CAR-T cell therapy could lead to clinical cure in some R/R LBCL patients.^20^^,^^21^ Results of ZUMA-7^22^ and TRANSFORM^23^ studies show that the efficacy of CAR-T cells against LBCL is superior to standard treatments including auto-HSCT. Since the efficacy of CAR-T cell therapy in second line was confirmed, the role of CAR-T in first-line therapy remains to be proved. ZUMA-12 is the first exploration for high-risk LBCL patients after two cycles of incomplete immunochemotherapy. Thirty-seven patients were included in the initial efficacy analysis and the ORR was 89% and the CR rate was 78%.^24^ The results of the ZUMA-12 study have shown good efficacy and safety, but still need further exploration to clarify the role of axi-cel as first-line therapy compared with standard immunochemotherapy.

In a single-center, phase 2a study, Tisagenlecleucel (a CD19 targeted CAR-T cell therapy developed by Norvartis) was tested in adult relapsed or refractory DLBCL who were ineligible for or had disease progression after autologous HSCT. The best ORR was 52%; 40% of the patients had complete responses, and 12% had partial responses. At 12 months after the initial response, the rate of relapse-free survival was estimated to be 65%. The most common grade 3 or 4 adverse events of special interest included cytokine release syndrome (22%), neurologic events (12%), cytopenias lasting more than 28 days (32%), infections (20%), and febrile neutropenia (14%).^25^ Despite the sensitivity of patients with relapsed/refractory lymphoma to chemotherapy is generally poor, there are still some patients who achieved CR. The JULIET study explored CAR-T cell bridging chemotherapy for such patients with tisa-cel. The expansion and pharmacokinetic characteristics of CAR-T cells were similar to those patients with incomplete response. The 3-month CR rate was 100%, the median follow-up time was 14.5 months, and the 1-year progression-free survival rate was 71.4%.^26^^,^^27^

Lisocabtagene maraleucel (a CD19 targeted CAR-T cell therapy developed by Juno Therapeutics) was tested in an open-label, phase 2 study (PILOT) as second-line therapy in 61 adults with relapsed or refractory large B cell lymphoma who were not intended for HSCT. The ORR was 80% while 48% of patients achieved CR, 72% of patients had grade ≥3 treatment-emergent AEs, 40% of which were cytopenias, 20% had cytokine release syndrome, and 12% had neurological events.^28^

Another multi-center study reported CAR-T cell therapy for R/R DLBCL patients with different remission states, and the results showed that the 1-year event-free survival rates were 59.6% for no disease patients, 11% for patients with focal lesions, at a median follow-up of 16 months. The 1-year OS rate was 81.3% and 59%, respectively, the 100-day CR rate of the focal lesions group was 78.8%.^29^ In summary, for salvage/bridging patients with CR after treatment, CAR-T cell therapy can be used as consolidation therapy to reduce the risk of lymphoma recurrence, and the safety is acceptable.

As CD19 antigen loss has been recognized as a key reason for the disease relapse of CD19 targeted CAR-T cell therapy in B cell malignancies, dual antigen targeting strategies were developed. CD20, a B cell antigen frequently expressed by NHL was utilized in combination with CD19. In a phase 1 dose escalation and expansion trial, bispecific anti-CD20, anti-CD19 CAR-T cells were tested in R/R NHL. The results showed that 82% of patients achieved an overall response at day 28, 64% had a complete response, and 18% had a partial response. Grade 3–4 cytokine release syndrome occurred in one (5%) patient, and grade 3–4 neurotoxicity occurred in 14% of patients.^30^ In another single-arm, phase 1–2 trial, 58 patients with aggressive DLBCL (24 with high tumor burden) were treated with tandem CD19/CD20 CAR therapy. The results showed that the best ORR was 78%, the median progression-free survival was 27.6 months (11 to not reached). Cytokine release syndrome occurred in 70% of patients and cell-related encephalopathy syndrome (CRES) occurred in 2% of patients.^31^^,^^32^ Collectively, these findings highlight the efficacy and safety of CD19, CD20 dual targeting strategy in B cell lymphoma; however, its long-term benefits await further validation.

### CAR-T cell exhaustion in lymphoma

Unlike leukemia, lymphomas are solid cancers that can form tumor lesions in organs and tissues and create an immune-suppressive TME that primarily consists of tumor cells, immune cells, and cytokines.^11^^,^^33^ Zachary et al. used single-cell RNA sequencing and protein surface marker profiling to analyze CAR-T cells pre- and post-infusion in patients with NHL and found that the evolution of CAR-T cells toward a non-proliferative, highly differentiated, and exhausted state, with an enriched exhaustion profile in CAR-T cells of patients with poor response marked by TIGIT expression.^34^ Katia and colleagues found CAR-T cells from patients who did not respond to therapy showed reduced ability to expand *in vitro* and decreased interleukin (IL)-2 secretion, indicating they are functionally deficient.^35^ Zhu and colleagues reported that CAR-T cells showed the characteristics of exhaustion after infiltration into tumor tissue, and the durability of effector function is impaired, resulting in limited therapeutic effect in lymphoma.^36^ Of note, Nathalie et al. evaluated the TME of 135 pre-treatment and post-treatment tumor biopsies taken from 51 patients in the ZUMA-1 phase 2 trial, and revealed that immune TME features rapidly changed after axi-cel infusion and high density of PD-1+LAG-3+ T cells in pre-treatment TME associated with overall response. More importantly, they also found systemic T cell exhaustion, including tumor-infiltrating lymphocytes and circulating CAR-T cells, associates with a lack of durable response to cell therapy.^37^ Collectively, these findings highlight the occurrence of CAR-T exhaustion when treating lymphoma patients and suggest exhaustion in CAR-T cell treatment of lymphoma patients.

## Mechanisms of CAR-T cell exhaustion

### Correlation of T cell exhaustion with T cell differentiation

**Figure 1:**
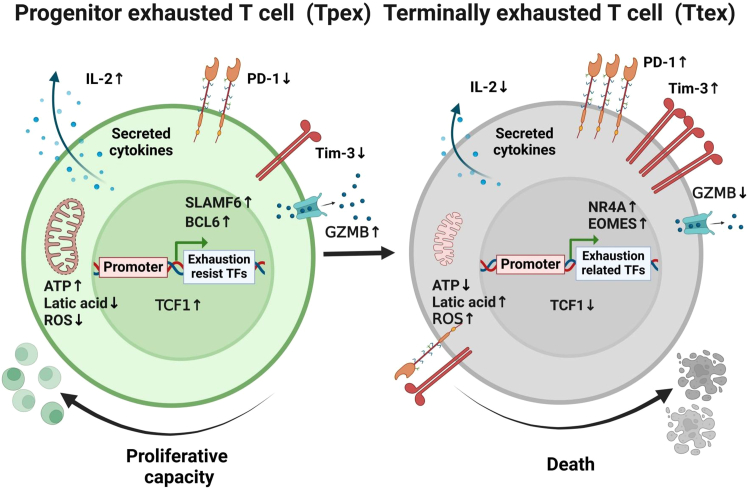
Correlation of T cell exhaustion with T cell differentiation

After thymic development, immature T cells were differentiated into single positive CD4 or CD8 mature T cells and entered into periphery tissues and the lymphoid system. These cells are naive to antigen exposure. When they encounter antigen stimulation, naive T cells can differentiate into memory or effector T cells.^38^^,^^39^ TCF1, a transcription factor, maintains the naive/memory state of T cells by promoting their capacity to proliferate and self-renew.^40^^,^^41^ Recent studies have found that T cell exhaustion is not a fixed state, but rather a dynamic process from “progenitor exhaustion” to “terminal exhaustion” during the progression of chronic infections or cancers. This process involves precursor exhausted T cells (Tpex) and terminally exhausted T cells (Ttex), which differ in the expression level of TCF1 and the proliferative capacity.^4^^,^^42^ Studies have shown that CD8+ Tpex cells not only rely on the transcription factor TCF1 but also express SLAMF6 and BCL6.^43^^,^^44^ Tpex cells have certain stem cell and memory cell-like characteristics, and have the ability to proliferate, self-renew, and generate terminally differentiated PD-1+ TCF1− GZMB+ cells. Tpex cell populations can expand and produce effector T cells when chronically infected by viruses or when blocking PD-1 immune checkpoints.^43^^,^^45^ When transplanted into infected or tumor-tolerant mice, Tpex cells can proliferate and produce Ttex cells, but if the Ttex cells are transplanted into the mice, the cells can only expand a small amount and also maintain the high expression of both PD-1 and Tim-3 (Figure 1).^44^ It is considered that the proliferation of TCF1+ Tpex cells population and the induction of effector functions may be a reaction after the blockage of immune checkpoints, but the mechanism of the above-mentioned reaction is still not fully understood. Based on the above findings from T cell biology, it is considered that exhaustion may be a process that can be parallel to conventional differentiation of T cells, and that T cells in any differentiated state can be induced to the exhaustion state and retain the characteristics of exhaustion in their next generation.^3^

Data from animal experiments demonstrate CAR-T cell exhaustion in the mouse model of multiple types of cancers. One study found that in tumor-infiltrating CAR-T cells, there were CD8+ CAR+ PD-1+ TIM3+ cells, and exhaustion-related transcriptional factor TOX was highly expressed.^46^ Wang et al. utilized single-cell transcriptomic sequencing and confirmed that antigen-specific CAR-T cells showed exhaustion characteristics.^47^ Prativa et al. constructed the mathematical analysis model and showed that with the increase of tumor antigen level, the level of CAR-T exhaustion also accelerated significantly.^48^ Besides, more studies have revealed the role of key factors including TOX, NR4A, TET2, and BATF in regulating the dynamic changes of CAR-T cell exhaustion, which will be more deeply discussed in the next section.

### Epigenetics of T cell exhaustion

Recent studies reported the utilization of ATAC-seq analysis to study exhausted T cell subsets.^4^^,^^49^^,^^50^ Tpex cells showed increased accessibility of TCF-1 and BACH2, while Ttex cells showed enrichments in NR4A and EOMES.^49^ It was found that exhausted T cells were epigenetically distinct from effector T cells, with nearly 5,000 differentially accessible regulatory elements. Similarly, H3K27ac chromatin immunoprecipitation sequencing (ChIP-seq) analysis demonstrated that Tpex cells exhibited the most distinct active enhancer landscape compared with Ttex cells.^50^ HA-28z is a second-generation anti-GD2 CAR, which forms clusters and causes spontaneous exhaustion when cultured *in vitro*; therefore, it was utilized as a classic exhausted CAR-T cell model. H3K27ac HiChIP analysis found that although there were only minimal changes in chromatin accessibility, differential chromatin looping was identified in HA-28z CAR-T cells, suggesting a further layer of exhausted T cells that should be investigated in future studies.^51^ Collectively, these findings described a distinct transcriptional and epigenetic feature for exhausted T cells and was stably maintained by certain molecular mechanisms.

### Aberrant metabolism contributes to exhaustion

Extensive alterations of metabolic profile have also been reported for T cell exhaustion. Although the exact mechanism is unknown, in the TME, CD8+ T cells face fierce competition for nutrients and are exposed to large amounts of toxic by-products. These will suppress the immune response and have a profound impact on T cell functionality, resulting in the functional exhaustion.^52^ Specifically, glucose,^53^ amino acids,^54^ and other nutrients substance deprivation as well as a low oxygen state^55^ have been found to accelerate CD8+ T cell exhaustion.^56^ Other metabolites, such as high extracellular lactate levels, were found to block CD8+ T cell proliferation in solid cancers. Besides, lipid metabolism may contribute to CD8+ T cell exhaustion, and sterols play a favorable role in T cell immune responses, suggesting that lipid accumulation and catabolism maybe targets to reverse CD8+ T cell antitumor response.^57^^,^^58^ These findings suggest metabolic stress may constrain T cell function and contribute to T cell exhaustion.

## Strategies to overcome CAR-T cell exhaustion

**Figure 2:**
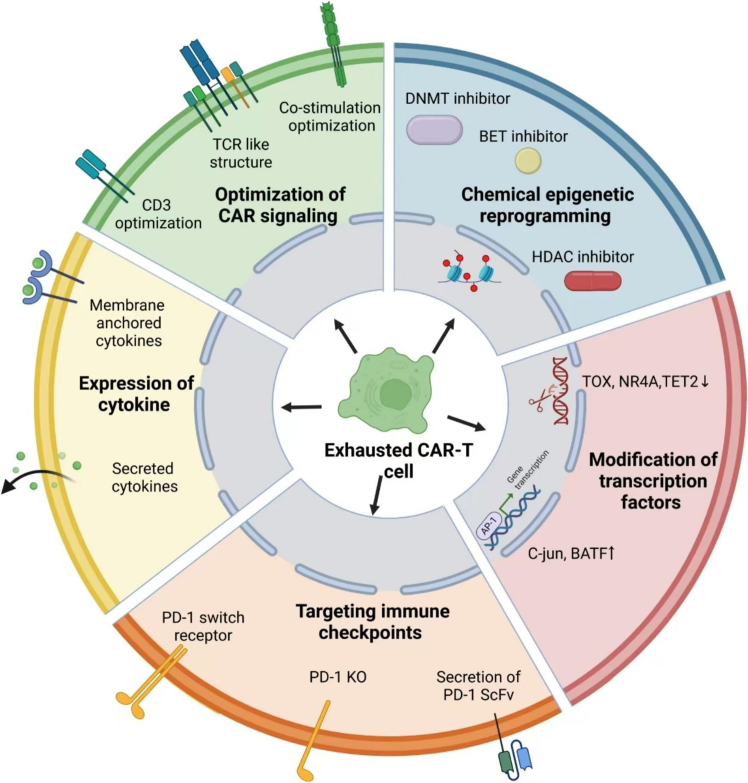
Strategies to overcome CAR-T cell exhaustion

The above findings strongly suggested that exhaustion is critical for the inferior antitumor activity of CAR-T cells. In recent years, numerous studies explored the potential strategies to overcome exhaustion in CAR-T cells, or at least attempted to ameliorate the CAR-T exhaustion extent. These contents are summarized in Figure 2.

### Optimization of CAR signaling

It has been accepted that CD3 and co-stimulation signals used in CAR endo-domain have a profound impact on CAR-T cell exhaustion states.

A recent study found that CARs containing CD3δ, CD3ε, or CD3γ cytoplasmic domains outperformed the conventional CD3ζ *in vivo*. Transcriptomic and proteomic analysis revealed the differences in the activation potential, metabolism, as well as T cell exhaustion state that mechanistically explain the enhanced antitumor performance.^59^ Another previous study discovered that the substitution of CD3ε with CD3ζ reduced cytokine production and promoted CAR-T persistence via p85 recruitment.^60^ CD28 and 4-1BB, two co-stimulation molecules that generally are used in the second-generation CAR, showed contrasting role to induce the exhaustion.^61^^,^^62^ Despite 4-1BB showing better ability to ameliorate exhaustion than CD28, the optimization for the redundancy of CD28 and CD3ζ signaling proved to be superior than 4-1BB incorporation.^63^ Based on this hypothesis, Feucht and colleagues designed 1XX CAR-T cells that persisted longer *in vivo* and had a higher percentage of central memory cells.^63^ Since the structure of conventional CAR is very much different from the natural structure of TCRs, some researchers then conceived TCRs like CAR structure. Liu et al. synthesized a double-chain chimeric receptor, termed as synthetic T cell receptor and antigen receptor (STAR), which incorporates antigen-recognition domain of antibody and constant regions of TCR that engage endogenous CD3 signaling machinery. They found that STAR-T cells prominently outperformed BBz CAR-T cells and generated better or equi-potent antitumor effects to 28z CAR-T cells without causing notable toxicity^64^ and the phase 1 clinical trial (NCT03953599) of this therapy for refractory and relapsed B cell acute lymphoblastic leukemia (B-ALL) patients is going on.^65^ Patrick and colleagues constructed another synthetic T cell receptor fusion construct (TRuC) receptor engaging the complete TCR for potent antitumor response.^66^^,^^67^ A phase 1 trial using this strategy was achieved and the result showed that substantial solid cancer patients responded to therapy.^68^ One *Nature* paper reported that targeting a CAR to the TRAC locus with CRISPR-Cas9 enhances tumor rejection.^69^ Dasatinib treatment in CAR-T cells decreases tonic signaling and also reduces CAR-T cell exhaustion.^70^ Collectively, these findings suggested the optimization of CAR signaling is critical for the improvement of CAR-T cell persistence and amelioration of CAR-T cell exhaustion.

### Integration of cytokines

Cytokines that were generally used to promote T cell expansion *in vitro* were subject to intensive study to improve CAR-T cell persistence and ameliorate exhaustion in pre-clinical models.^71^^,^^72^^,^^73^^,^^74^^,^^75^ However, release of these cytokines into the extracellular space may cause immune toxicities. To solve this problem, Thomas and colleagues constructed a constitutively signaling cytokine receptor, C7R, which potently triggers the IL-7 signaling axis but is unresponsive to extracellular cytokines for CAR-T cells.^76^ Jiang et al. incorporated GP130 signaling motifs in CAR and overcome the toxicity caused by the overexpression of interleukin,^77^ while Yuki et al. constructed a novel CAR containing a JAK-STAT signaling domain and found it mediates superior antitumor effects.^78^ Besides, membrane-anchored cytokines were recently reported to be superior than the secreted form in their abilities to enhance tumor targeting and reduce systemic toxicity.^79^^,^^80^ Of note, IL-10, a cytokine that was originally believed to suppress immune responses was recently found to enhance the function of CAR-T cells by improving the metabolic fitness and reducing exhaustion extent.^81^ Altogether, these findings suggest the potential of using cytokines to ameliorate the exhaustion of CAR-T cells, which is of great potential to be translated into clinics against lymphoma (Table 1).

**Table 1:** The functions for the transcription factors and cytokines that are involved in CAR-T cell modification

IL-2 overexpression	Promote proliferation and exhaustion
IL-7 overexpression	Promote proliferation and Reducing exhaustion
IL-15 overexpression	Promote proliferation and Reducing exhaustion
IL-21 overexpression	Promote effector function and Reducing exhaustion
IL-12 overexpression	Promote IFN-gamma secretion, but cause toxicity
IL-10 overexpression	Reducing exhaustion
TOX/TOX2 knockout	Resistant exhaustion
NR4A knockout	Resistant exhaustion
BATF knockout	Resistant exhaustion
TET2 knockout	Resistant exhaustion

### Approaches to compromise the function of immune checkpoint molecules

Given that immune checkpoint (IC) molecules including PD-1 and CTLA4 are expressed on activated and exhausted CAR-T cells, while their ligands are generally expressed by tumor cells or other infiltrating immune cells in the TME, therefore studies have tested the strategies that compromise the functions of these checkpoints. These include the simple combination of IC antibodies with CAR-T,^82^^,^^83^^,^^84^ the overexpression of IC blocking antibodies,^85^^,^^86^ knockout or knocking down genes of ICs in CAR-T cells,^87^^,^^88^^,^^89^^,^^90^ and design of dominate negative receptors (DNRs)^91^ to block the signal of ICs or chimeric switch receptors (CSRs)^90^^,^^92^ to reverse their immune-suppressive functions into immune activating functions. However, some studies found that ICs like PD-1 or PD-L1 may also have positive influence for T cell functionality,^93^^,^^94^ hence, the comprehensive effect for the blockage of ICs in CAR-T cells remains elusive.

### Modification of transcription factor and epigenetic reprogramming

Several studies have attempted to knock out those key transcriptional factors that maintain the CAR-T cell exhaustion or to overexpress TFs that promote T cell persistence to enhance their activity against solid cancers. One study reported that both TOX and TOX2-deficient CAR-T were able to suppress tumor growth and prolong survival of tumor-bearing mice.^46^ Besides, it was reported that CAR-T cells lacking all three NR4A transcription factors (NR4A triple knockout) promoted tumor regression and prolonged the survival of tumor-bearing mice. NR4A triple knockout CAR tumor-infiltrating lymphocytes displayed phenotypes and gene expression profiles characteristic of CD8+ effector T cells, and chromatin regions uniquely accessible in NR4A triple knockout CAR tumor-infiltrating lymphocytes were enriched for binding motifs for NF-κB and AP-1; both are transcription factors involved in activation of T cells.^95^ Besides, Zhang et al. reported the depletion of BATF in CAR-T cells enhances antitumor activity by inducing resistance against exhaustion and formation of central memory cells.^96^ Moreover, in a clinical case, lentiviral vector-mediated insertion of the CAR transgene disrupted the methylcytosine dioxygenase TET2 gene and induced a drastic expansion in patients,^97^ and subsequent studies utilized TET2 disruption to render CAR-T cells resistant to exhaustion. However, the safety of TET2 disruption has been caught into question, as Nayan et al. found that TET2 guards against unchecked BATF3-induced CAR-T cell expansion, and its disruption would induce genomic instability of CAR-T cells.^98^

A study from Rachel and colleagues found that CAR-T cells engineered to overexpress the canonical AP-1 factor c-Jun have enhanced expansion potential, increased functional capacity, diminished terminal differentiation, and improved antitumor potency in five different mouse tumor models and concluded that a functional deficiency in c-Jun mediates dysfunction in exhausted human T cells, and the overexpression of c-Jun renders CAR-T cells resistant to exhaustion.^99^ Hyungseok et al. found that overexpression of BATF in CD8+ T cells expressing a CAR promoted the survival and expansion of tumor-infiltrating CAR-T cells, increased the production of effector cytokines, decreased the expression of inhibitory receptors and the exhaustion-associated transcription factor TOX, and supported the generation of long-lived memory T cells that controlled tumor recurrence.^100^ Besides, other transcription factors like FOXP1, KLF2, and Foxo1 were also reported to play a critical role in affecting the metabolism and memory formation and finally regulate CAR-T cell exhaustion.^101^^,^^102^

Modifying epigenetic marks through drugs or gene editing may “reset” exhausted T cells by altering transcription programs associated with effector function maintenance. One study reported that low-dose decitabine promotes the clonal expansion and cytolytic activity of progenitor exhausted T cells and restrained CD8+ T cell terminal differentiation.^103^ Hence, low-dose decitabine priming is able to endow CAR-T cells with persistent antitumor potential via epigenetic reprogramming.^104^ Zhong et al. reported the use of JQ1, a chemical drug candidate that inhibits BET bromodomain protein was able to rescue PD-1-mediated T cell exhaustion in acute myeloid leukemia.^105^ Collectively, these results highlight the importance of modifying epigenetics in CAR-T cells to overcome exhaustion.

### Conclusion

In summary, CAR-T therapy has achieved great successes in the clinical treatment of lymphoma patients, while the clinical success is dampened by instances of uncompleted remission and early relapsed patients. There are two main reasons that are considered to be responsible for this: one key mechanism is the loss of target antigens, another key mechanism is T cell exhaustion, which is the focus of this review. A variety of modifications to CAR-T cells, ranging from signaling, cytokines, checkpoint molecules, and transcription factors have been shown to potentially alleviate or prevent T cell exhaustion. More importantly, many strategies are being translated into the clinic, and these upcoming clinical studies will provide us with more information on how best to improve the therapeutic efficacy of CAR-T cells against lymphoma.

## Acknowledgments

We thank Jinghong Chen and Diya Cai from the Department of Hematology and Oncology, Shenzhen University General Hospital, for their technical support. This work is supported by 10.13039/501100017610Shenzhen Science and Technology Innovation Program (JCYJ20190808163601776, JCYJ20200109113810154, JCYJ20230807110406013), 10.13039/501100012151Sanming Project of Medicine in Shenzen Municipality (SZSM202111004), 10.13039/501100001809National Natural Science Foundation of China (82030076, 82070161), and Shenzhen medical research fund (C2301003).

## Author contributions

R.Z. and L.W. designed the manuscript. R.Z., J.X., Y.Z., and W.H. analyzed the data and wrote the manuscript. J.X., L.Y., and L.W. revised the manuscript. All authors read and approved the manuscript.

## Declaration of interests

The authors declare that the research was conducted in the absence of any commercial or financial relationships that could be construed as a potential conflict of interest.

## References

[1] Gumber D., Wang L.D. (2022). Improving CAR-T immunotherapy: Overcoming the challenges of T cell exhaustion. EBioMedicine.

[2] Kasakovski D., Xu L., Li Y. (2018). T cell senescence and CAR-T cell exhaustion in hematological malignancies. J. Hematol. Oncol..

[3] Blank C.U., Haining W.N., Held W., Hogan P.G., Kallies A., Lugli E., Lynn R.C., Philip M., Rao A., Restifo N.P. (2019). Defining ‘T cell exhaustion’. Nat. Rev. Immunol..

[4] Beltra J.C., Manne S., Abdel-Hakeem M.S., Kurachi M., Giles J.R., Chen Z., Casella V., Ngiow S.F., Khan O., Huang Y.J. (2020). Developmental Relationships of Four Exhausted CD8(+) T Cell Subsets Reveals Underlying Transcriptional and Epigenetic Landscape Control Mechanisms. Immunity.

[5] Chihara N., Madi A., Kondo T., Zhang H., Acharya N., Singer M., Nyman J., Marjanovic N.D., Kowalczyk M.S., Wang C. (2018). Induction and transcriptional regulation of the co-inhibitory gene module in T cells. Nature.

[6] Collins M.H., Henderson A.J. (2014). Transcriptional regulation and T cell exhaustion. Curr. Opin. HIV AIDS.

[7] Westin J., Sehn L.H. (2022). CAR T cells as a second-line therapy for large B-cell lymphoma: a paradigm shift?. Blood.

[8] Haslauer T., Greil R., Zaborsky N., Geisberger R. (2021). CAR T-Cell Therapy in Hematological Malignancies. Int. J. Mol. Sci..

[9] Ramos C.A., Heslop H.E., Brenner M.K. (2016). CAR-T Cell Therapy for Lymphoma. Annu. Rev. Med..

[10] Hopken U.E., Rehm A. (2019). Targeting the Tumor Microenvironment of Leukemia and Lymphoma. Trends Cancer.

[11] Cai F., Zhang J., Gao H., Shen H. (2024). Tumor microenvironment and CAR-T cell immunotherapy in B-cell lymphoma. Eur. J. Haematol..

[12] Shah N.N., Fry T.J. (2019). Mechanisms of resistance to CAR T cell therapy. Nat. Rev. Clin. Oncol..

[13] Liu Y., Zhou X., Wang X. (2021). Targeting the tumor microenvironment in B-cell lymphoma: challenges and opportunities. J. Hematol. Oncol..

[14] Sehn L.H., Salles G. (2021). Diffuse Large B-Cell Lymphoma. N. Engl. J. Med..

[15] Liu W., Qi J., Liu J., Song Y., Wang L., Zhou M., Ma J., Zhu J. (2022). Mortality Rate of Lymphoma in China, 2013-2020. Front. Oncol..

[16] Jiang M., Bennani N.N., Feldman A.L. (2017). Lymphoma classification update: T-cell lymphomas, Hodgkin lymphomas, and histiocytic/dendritic cell neoplasms. Expert Rev. Hematol..

[17] de Leval L., Jaffe E.S. (2020). Lymphoma Classification. Cancer J..

[18] Nastoupil L.J., Bartlett N.L. (2023). Navigating the Evolving Treatment Landscape of Diffuse Large B-Cell Lymphoma. J. Clin. Oncol..

[19] Armitage J.O., Gascoyne R.D., Lunning M.A., Cavalli F. (2017). Non-Hodgkin lymphoma. Lancet.

[20] Locke F.L., Ghobadi A., Jacobson C.A., Miklos D.B., Lekakis L.J., Oluwole O.O., Lin Y., Braunschweig I., Hill B.T., Timmerman J.M. (2019). Long-term safety and activity of axicabtagene ciloleucel in refractory large B-cell lymphoma (ZUMA-1): a single-arm, multicentre, phase 1-2 trial. Lancet Oncol..

[21] Neelapu S.S., Jacobson C.A., Ghobadi A., Miklos D.B., Lekakis L.J., Oluwole O.O., Lin Y., Braunschweig I., Hill B.T., Timmerman J.M. (2023). Five-year follow-up of ZUMA-1 supports the curative potential of axicabtagene ciloleucel in refractory large B-cell lymphoma. Blood.

[22] Locke F.L., Miklos D.B., Jacobson C.A., Perales M.A., Kersten M.J., Oluwole O.O., Ghobadi A., Rapoport A.P., McGuirk J., Pagel J.M. (2022). Axicabtagene Ciloleucel as Second-Line Therapy for Large B-Cell Lymphoma. N. Engl. J. Med..

[23] Kamdar M., Solomon S.R., Arnason J., Johnston P.B., Glass B., Bachanova V., Ibrahimi S., Mielke S., Mutsaers P., Hernandez-Ilizaliturri F. (2022). Lisocabtagene maraleucel versus standard of care with salvage chemotherapy followed by autologous stem cell transplantation as second-line treatment in patients with relapsed or refractory large B-cell lymphoma (TRANSFORM): results from an interim analysis of an open-label, randomised, phase 3 trial. Lancet.

[24] Neelapu S.S., Dickinson M., Munoz J., Ulrickson M.L., Thieblemont C., Oluwole O.O., Herrera A.F., Ujjani C.S., Lin Y., Riedell P.A. (2022). Axicabtagene ciloleucel as first-line therapy in high-risk large B-cell lymphoma: the phase 2 ZUMA-12 trial. Nat. Med..

[25] Maude S.L., Laetsch T.W., Buechner J., Rives S., Boyer M., Bittencourt H., Bader P., Verneris M.R., Stefanski H.E., Myers G.D. (2018). Tisagenlecleucel in Children and Young Adults with B-Cell Lymphoblastic Leukemia. N. Engl. J. Med..

[26] Schuster S.J., Tam C.S., Borchmann P., Worel N., McGuirk J.P., Holte H., Waller E.K., Jaglowski S., Bishop M.R., Damon L.E. (2021). Long-term clinical outcomes of tisagenlecleucel in patients with relapsed or refractory aggressive B-cell lymphomas (JULIET): a multicentre, open-label, single-arm, phase 2 study. Lancet Oncol..

[27] Westin J.R., Kersten M.J., Salles G., Abramson J.S., Schuster S.J., Locke F.L., Andreadis C. (2021). Efficacy and safety of CD19-directed CAR-T cell therapies in patients with relapsed/refractory aggressive B-cell lymphomas: Observations from the JULIET, ZUMA-1, and TRANSCEND trials. Am. J. Hematol..

[28] Sehgal A., Hoda D., Riedell P.A., Ghosh N., Hamadani M., Hildebrandt G.C., Godwin J.E., Reagan P.M., Wagner-Johnston N., Essell J. (2022). Lisocabtagene maraleucel as second-line therapy in adults with relapsed or refractory large B-cell lymphoma who were not intended for haematopoietic stem cell transplantation (PILOT): an open-label, phase 2 study. Lancet Oncol..

[29] Wudhikarn K., Tomas A.A., Flynn J.R., Devlin S.M., Brower J., Bachanova V., Nastoupil L.J., McGuirk J.P., Maziarz R.T., Oluwole O.O. (2023). Low toxicity and excellent outcomes in patients with DLBCL without residual lymphoma at the time of CD19 CAR T-cell therapy. Blood Adv..

[30] Shah N.N., Johnson B.D., Schneider D., Zhu F., Szabo A., Keever-Taylor C.A., Krueger W., Worden A.A., Kadan M.J., Yim S. (2020). Bispecific anti-CD20, anti-CD19 CAR T cells for relapsed B cell malignancies: a phase 1 dose escalation and expansion trial. Nat. Med..

[31] Zhang Y., Wang Y., Liu Y., Tong C., Wang C., Guo Y., Ti D., Yang Q., Qiao S., Wu Z., Han W. (2022). Long-term activity of tandem CD19/CD20 CAR therapy in refractory/relapsed B-cell lymphoma: a single-arm, phase 1-2 trial. Leukemia.

[32] Tong C., Zhang Y., Liu Y., Ji X., Zhang W., Guo Y., Han X., Ti D., Dai H., Wang C. (2020). Optimized tandem CD19/CD20 CAR-engineered T cells in refractory/relapsed B-cell lymphoma. Blood.

[33] Dreyzin A., Rankin A.W., Luciani K., Gavrilova T., Shah N.N. (2024). Overcoming the challenges of primary resistance and relapse after CAR-T cell therapy. Expert Rev. Clin. Immunol..

[34] Jackson Z., Hong C., Schauner R., Dropulic B., Caimi P.F., de Lima M., Giraudo M.F., Gupta K., Reese J.S., Hwang T.H., Wald D.N. (2022). Sequential Single-Cell Transcriptional and Protein Marker Profiling Reveals TIGIT as a Marker of CD19 CAR-T Cell Dysfunction in Patients with Non-Hodgkin Lymphoma. Cancer Discov..

[35] Beider K., Itzhaki O., Schachter J., Grushchenko-Polaq A.H., Voevoda-Dimenshtein V., Rosenberg E., Ostrovsky O., Devillers O., Shapira Frommer R., Zeltzer L.A. (2022). Molecular and Functional Signatures Associated with CAR T Cell Exhaustion and Impaired Clinical Response in Patients with B Cell Malignancies. Cells.

[36] Zhu X., Chen J., Li W., Xu Y., Shan J., Hong J., Zhao Y., Xu H., Ma J., Shen J., Qian C. (2024). Hypoxia-Responsive CAR-T Cells Exhibit Reduced Exhaustion and Enhanced Efficacy in Solid Tumors. Cancer Res..

[37] Scholler N., Perbost R., Locke F.L., Jain M.D., Turcan S., Danan C., Chang E.C., Neelapu S.S., Miklos D.B., Jacobson C.A. (2022). Tumor immune contexture is a determinant of anti-CD19 CAR T cell efficacy in large B cell lymphoma. Nat. Med..

[38] Kumar B.V., Connors T.J., Farber D.L. (2018). Human T Cell Development, Localization, and Function throughout Life. Immunity.

[39] Yu Q., Sharma A., Sen J.M. (2010). TCF1 and beta-catenin regulate T cell development and function. Immunol. Res..

[40] Gounari F., Khazaie K. (2022). TCF-1: a maverick in T cell development and function. Nat. Immunol..

[41] Zhao X., Shan Q., Xue H.H. (2022). TCF1 in T cell immunity: a broadened frontier. Nat. Rev. Immunol..

[42] Im S.J., Hashimoto M., Gerner M.Y., Lee J., Kissick H.T., Burger M.C., Shan Q., Hale J.S., Lee J., Nasti T.H. (2016). Defining CD8+ T cells that provide the proliferative burst after PD-1 therapy. Nature.

[43] Utzschneider D.T., Charmoy M., Chennupati V., Pousse L., Ferreira D.P., Calderon-Copete S., Danilo M., Alfei F., Hofmann M., Wieland D. (2016). T Cell Factor 1-Expressing Memory-like CD8(+) T Cells Sustain the Immune Response to Chronic Viral Infections. Immunity.

[44] Siddiqui I., Schaeuble K., Chennupati V., Fuertes Marraco S.A., Calderon-Copete S., Pais Ferreira D., Carmona S.J., Scarpellino L., Gfeller D., Pradervand S. (2019). Intratumoral Tcf1(+)PD-1(+)CD8(+) T Cells with Stem-like Properties Promote Tumor Control in Response to Vaccination and Checkpoint Blockade Immunotherapy. Immunity.

[45] Wu T., Ji Y., Moseman E.A., Xu H.C., Manglani M., Kirby M., Anderson S.M., Handon R., Kenyon E., Elkahloun A. (2016). The TCF1-Bcl6 axis counteracts type I interferon to repress exhaustion and maintain T cell stemness. Sci. Immunol..

[46] Seo H., Chen J., González-Avalos E., Samaniego-Castruita D., Das A., Wang Y.H., López-Moyado I.F., Georges R.O., Zhang W., Onodera A. (2019). TOX and TOX2 transcription factors cooperate with NR4A transcription factors to impose CD8(+) T cell exhaustion. Proc. Natl. Acad. Sci. USA.

[47] Wang X., Peticone C., Kotsopoulou E., Göttgens B., Calero-Nieto F.J. (2021). Single-cell transcriptome analysis of CAR T-cell products reveals subpopulations, stimulation, and exhaustion signatures. OncoImmunology.

[48] Sahoo P., Yang X., Abler D., Maestrini D., Adhikarla V., Frankhouser D., Cho H., Machuca V., Wang D., Barish M. (2020). Mathematical deconvolution of CAR T-cell proliferation and exhaustion from real-time killing assay data. J. R. Soc. Interface.

[49] Yao C., Lou G., Sun H.W., Zhu Z., Sun Y., Chen Z., Chauss D., Moseman E.A., Cheng J., D'Antonio M.A. (2021). BACH2 enforces the transcriptional and epigenetic programs of stem-like CD8(+) T cells. Nat. Immunol..

[50] Utzschneider D.T., Gabriel S.S., Chisanga D., Gloury R., Gubser P.M., Vasanthakumar A., Shi W., Kallies A. (2020). Early precursor T cells establish and propagate T cell exhaustion in chronic infection. Nat. Immunol..

[51] Gennert D.G., Lynn R.C., Granja J.M., Weber E.W., Mumbach M.R., Zhao Y., Duren Z., Sotillo E., Greenleaf W.J., Wong W.H. (2021). Dynamic chromatin regulatory landscape of human CAR T cell exhaustion. Proc. Natl. Acad. Sci. USA.

[52] Chang C.H., Qiu J., O'Sullivan D., Buck M.D., Noguchi T., Curtis J.D., Chen Q., Gindin M., Gubin M.M., van der Windt G.J.W. (2015). Metabolic Competition in the Tumor Microenvironment Is a Driver of Cancer Progression. Cell.

[53] Chang C.H., Curtis J.D., Maggi L.B., Faubert B., Villarino A.V., O'Sullivan D., Huang S.C.C., van der Windt G.J.W., Blagih J., Qiu J. (2013). Posttranscriptional control of T cell effector function by aerobic glycolysis. Cell.

[54] Zhang L., Romero P. (2018). Metabolic Control of CD8(+) T Cell Fate Decisions and Antitumor Immunity. Trends Mol. Med..

[55] Schieber M., Chandel N.S. (2014). ROS function in redox signaling and oxidative stress. Curr. Biol..

[56] Jung J.G., Le A. (2021). Metabolism of Immune Cells in the Tumor Microenvironment. Adv. Exp. Med. Biol..

[57] Zhang Y., Kurupati R., Liu L., Zhou X.Y., Zhang G., Hudaihed A., Filisio F., Giles-Davis W., Xu X., Karakousis G.C. (2017). Enhancing CD8(+) T Cell Fatty Acid Catabolism within a Metabolically Challenging Tumor Microenvironment Increases the Efficacy of Melanoma Immunotherapy. Cancer Cell.

[58] Yang W., Bai Y., Xiong Y., Zhang J., Chen S., Zheng X., Meng X., Li L., Wang J., Xu C. (2016). Potentiating the antitumour response of CD8(+) T cells by modulating cholesterol metabolism. Nature.

[59] Velasco Cardenas R.M., Brandl S.M., Melendez A.V., Schlaak A.E., Buschky A., Peters T., Beier F., Serrels B., Taromi S., Raute K. (2023). Harnessing CD3 diversity to optimize CAR T cells. Nat. Immunol..

[60] Wu W., Zhou Q., Masubuchi T., Shi X., Li H., Xu X., Huang M., Meng L., He X., Zhu H. (2020). Multiple Signaling Roles of CD3epsilon and Its Application in CAR-T Cell Therapy. Cell.

[61] Long A.H., Haso W.M., Shern J.F., Wanhainen K.M., Murgai M., Ingaramo M., Smith J.P., Walker A.J., Kohler M.E., Venkateshwara V.R. (2015). 4-1BB costimulation ameliorates T cell exhaustion induced by tonic signaling of chimeric antigen receptors. Nat. Med..

[62] Cappell K.M., Kochenderfer J.N. (2021). A comparison of chimeric antigen receptors containing CD28 versus 4-1BB costimulatory domains. Nat. Rev. Clin. Oncol..

[63] Feucht J., Sun J., Eyquem J., Ho Y.J., Zhao Z., Leibold J., Dobrin A., Cabriolu A., Hamieh M., Sadelain M. (2019). Calibration of CAR activation potential directs alternative T cell fates and therapeutic potency. Nat. Med..

[64] Liu Y., Liu G., Wang J., Zheng Z.Y., Jia L., Rui W., Huang D., Zhou Z.X., Zhou L., Wu X. (2021). Chimeric STAR receptors using TCR machinery mediate robust responses against solid tumors. Sci. Transl. Med..

[65] Wang J., Zhang X., Zhou Z., Liu Y., Yu L., Jia L., Yang J., Li J., Yu H., Li W. (2022). A novel adoptive synthetic TCR and antigen receptor (STAR) T-Cell therapy for B-Cell acute lymphoblastic leukemia. Am. J. Hematol..

[66] Ding J., Guyette S., Schrand B., Geirut J., Horton H., Guo G., Delgoffe G., Menk A., Baeuerle P.A., Hofmeister R., Tighe R. (2023). Mesothelin-targeting T cells bearing a novel T cell receptor fusion construct (TRuC) exhibit potent antitumor efficacy against solid tumors. OncoImmunology.

[67] Baeuerle P.A., Ding J., Patel E., Thorausch N., Horton H., Gierut J., Scarfo I., Choudhary R., Kiner O., Krishnamurthy J. (2019). Synthetic TRuC receptors engaging the complete T cell receptor for potent anti-tumor response. Nat. Commun..

[68] Hassan R., Butler M., O'Cearbhaill R.E., Oh D.Y., Johnson M., Zikaras K., Smalley M., Ross M., Tanyi J.L., Ghafoor A. (2023). Mesothelin-targeting T cell receptor fusion construct cell therapy in refractory solid tumors: phase 1/2 trial interim results. Nat. Med..

[69] Eyquem J., Mansilla-Soto J., Giavridis T., van der Stegen S.J.C., Hamieh M., Cunanan K.M., Odak A., Gönen M., Sadelain M. (2017). Targeting a CAR to the TRAC locus with CRISPR/Cas9 enhances tumour rejection. Nature.

[70] Zhang H., Hu Y., Shao M., Teng X., Jiang P., Wang X., Wang H., Cui J., Yu J., Liang Z. (2021). Dasatinib enhances anti-leukemia efficacy of chimeric antigen receptor T cells by inhibiting cell differentiation and exhaustion. J. Hematol. Oncol..

[71] Adachi K., Kano Y., Nagai T., Okuyama N., Sakoda Y., Tamada K. (2018). IL-7 and CCL19 expression in CAR-T cells improves immune cell infiltration and CAR-T cell survival in the tumor. Nat. Biotechnol..

[72] Alizadeh D., Wong R.A., Yang X., Wang D., Pecoraro J.R., Kuo C.F., Aguilar B., Qi Y., Ann D.K., Starr R. (2019). IL15 Enhances CAR-T Cell Antitumor Activity by Reducing mTORC1 Activity and Preserving Their Stem Cell Memory Phenotype. Cancer Immunol. Res..

[73] Lanitis E., Rota G., Kosti P., Ronet C., Spill A., Seijo B., Romero P., Dangaj D., Coukos G., Irving M. (2021). Optimized gene engineering of murine CAR-T cells reveals the beneficial effects of IL-15 coexpression. J. Exp. Med..

[74] Batra S.A., Rathi P., Guo L., Courtney A.N., Fleurence J., Balzeau J., Shaik R.S., Nguyen T.P., Wu M.F., Bulsara S. (2020). Glypican-3-Specific CAR T Cells Coexpressing IL15 and IL21 Have Superior Expansion and Antitumor Activity against Hepatocellular Carcinoma. Cancer Immunol. Res..

[75] Zhang Y., Zhang C., He M., Xing W., Hou R., Zhang H. (2024). Co-expression of IL-21-Enhanced NKG2D CAR-NK cell therapy for lung cancer. BMC Cancer.

[76] Shum T., Omer B., Tashiro H., Kruse R.L., Wagner D.L., Parikh K., Yi Z., Sauer T., Liu D., Parihar R. (2017). Constitutive Signaling from an Engineered IL7 Receptor Promotes Durable Tumor Elimination by Tumor-Redirected T Cells. Cancer Discov..

[77] Jiang Z., Liao R., Lv J., Li S., Zheng D., Qin L., Wu D., Chen S., Long Y., Wu Q. (2021). IL-6 trans-signaling promotes the expansion and anti-tumor activity of CAR T cells. Leukemia.

[78] Kagoya Y., Tanaka S., Guo T., Anczurowski M., Wang C.H., Saso K., Butler M.O., Minden M.D., Hirano N. (2018). A novel chimeric antigen receptor containing a JAK-STAT signaling domain mediates superior antitumor effects. Nat. Med..

[79] Nguyen R., Doubrovina E., Mousset C.M., Jin B.Y., Okada R., Zhang X., Clavel A., Reyes-Gonzalez J.M., Dyomin V., Diaz L. (2024). Cooperative Armoring of CAR and TCR T Cells by T Cell-Restricted IL15 and IL21 Universally Enhances Solid Tumor Efficacy. Clin. Cancer Res..

[80] Lee E.H.J., Murad J.P., Christian L., Gibson J., Yamaguchi Y., Cullen C., Gumber D., Park A.K., Young C., Monroy I. (2023). Antigen-dependent IL-12 signaling in CAR T cells promotes regional to systemic disease targeting. Nat. Commun..

[81] Zhao Y., Chen J., Andreatta M., Feng B., Xie Y.Q., Wenes M., Wang Y., Gao M., Hu X., Romero P. (2024). IL-10-expressing CAR T cells resist dysfunction and mediate durable clearance of solid tumors and metastases. Nat. Biotechnol..

[82] Xu J., Zhang Q., Tian K., Wang H., Yin H., Zheng J. (2018). Current status and future prospects of the strategy of combining CAR-T with PD-1 blockade for antitumor therapy (Review). Mol. Med. Rep..

[83] Adusumilli P.S., Zauderer M.G., Rivière I., Solomon S.B., Rusch V.W., O'Cearbhaill R.E., Zhu A., Cheema W., Chintala N.K., Halton E. (2021). A Phase I Trial of Regional Mesothelin-Targeted CAR T-cell Therapy in Patients with Malignant Pleural Disease, in Combination with the Anti-PD-1 Agent Pembrolizumab. Cancer Discov..

[84] Grosser R., Cherkassky L., Chintala N., Adusumilli P.S. (2019). Combination Immunotherapy with CAR T Cells and Checkpoint Blockade for the Treatment of Solid Tumors. Cancer Cell.

[85] Rafiq S., Yeku O.O., Jackson H.J., Purdon T.J., van Leeuwen D.G., Drakes D.J., Song M., Miele M.M., Li Z., Wang P. (2018). Targeted delivery of a PD-1-blocking scFv by CAR-T cells enhances anti-tumor efficacy in vivo. Nat. Biotechnol..

[86] Chen J., Zhu T., Jiang G., Zeng Q., Li Z., Huang X. (2023). Target delivery of a PD-1-TREM2 scFv by CAR-T cells enhances anti-tumor efficacy in colorectal cancer. Mol. Cancer.

[87] McGowan E., Lin Q., Ma G., Yin H., Chen S., Lin Y. (2020). PD-1 disrupted CAR-T cells in the treatment of solid tumors: Promises and challenges. Biomed. Pharmacother..

[88] Agarwal S., Aznar M.A., Rech A.J., Good C.R., Kuramitsu S., Da T., Gohil M., Chen L., Hong S.J.A., Ravikumar P. (2023). Deletion of the inhibitory co-receptor CTLA-4 enhances and invigorates chimeric antigen receptor T cells. Immunity.

[89] Lau E., Kwong G., Fowler T.W., Sun B.C., Donohoue P.D., Davis R.T., Bryan M., McCawley S., Clarke S.C., Williams C. (2023). Allogeneic chimeric antigen receptor-T cells with CRISPR-disrupted programmed death-1 checkpoint exhibit enhanced functional fitness. Cytotherapy.

[90] Lorenzini T., Cadilha B.L., Obeck H., Benmebarek M.R., Märkl F., Michaelides S., Strzalkowski T., Briukhovetska D., Müller P.J., Nandi S. (2023). Rational design of PD-1-CD28 immunostimulatory fusion proteins for CAR T cell therapy. Br. J. Cancer.

[91] Cherkassky L., Morello A., Villena-Vargas J., Feng Y., Dimitrov D.S., Jones D.R., Sadelain M., Adusumilli P.S. (2016). Human CAR T cells with cell-intrinsic PD-1 checkpoint blockade resist tumor-mediated inhibition. J. Clin. Investig..

[92] Chen C., Gu Y.M., Zhang F., Zhang Z.C., Zhang Y.T., He Y.D., Wang L., Zhou N., Tang F.T., Liu H.J., Li Y.M. (2021). Construction of PD1/CD28 chimeric-switch receptor enhances anti-tumor ability of c-Met CAR-T in gastric cancer. OncoImmunology.

[93] Wei J., Luo C., Wang Y., Guo Y., Dai H., Tong C., Ti D., Wu Z., Han W. (2019). PD-1 silencing impairs the anti-tumor function of chimeric antigen receptor modified T cells by inhibiting proliferation activity. J. Immunother. Cancer.

[94] Sailer C.J., Hong Y., Dahal A., Ryan A.T., Mir S., Gerber S.A., Reagan P.M., Kim M. (2023). PD-1(Hi) CAR-T cells provide superior protection against solid tumors. Front. Immunol..

[95] Chen J., López-Moyado I.F., Seo H., Lio C.W.J., Hempleman L.J., Sekiya T., Yoshimura A., Scott-Browne J.P., Rao A. (2019). NR4A transcription factors limit CAR T cell function in solid tumours. Nature.

[96] Zhang X., Zhang C., Qiao M., Cheng C., Tang N., Lu S., Sun W., Xu B., Cao Y., Wei X. (2022). Depletion of BATF in CAR-T cells enhances antitumor activity by inducing resistance against exhaustion and formation of central memory cells. Cancer Cell.

[97] Fraietta J.A., Nobles C.L., Sammons M.A., Lundh S., Carty S.A., Reich T.J., Cogdill A.P., Morrissette J.J.D., DeNizio J.E., Reddy S. (2018). Disruption of TET2 promotes the therapeutic efficacy of CD19-targeted T cells. Nature.

[98] Jain N., Zhao Z., Feucht J., Koche R., Iyer A., Dobrin A., Mansilla-Soto J., Yang J., Zhan Y., Lopez M. (2023). TET2 guards against unchecked BATF3-induced CAR T cell expansion. Nature.

[99] Lynn R.C., Weber E.W., Sotillo E., Gennert D., Xu P., Good Z., Anbunathan H., Lattin J., Jones R., Tieu V. (2019). c-Jun overexpression in CAR T cells induces exhaustion resistance. Nature.

[100] Seo H., González-Avalos E., Zhang W., Ramchandani P., Yang C., Lio C.W.J., Rao A., Hogan P.G. (2021). BATF and IRF4 cooperate to counter exhaustion in tumor-infiltrating CAR T cells. Nat. Immunol..

[101] Zhu Z., Lou G., Teng X.L., Wang H., Luo Y., Shi W., Yihunie K., Hao S., DeGolier K., Liao C. (2024). FOXP1 and KLF2 reciprocally regulate checkpoints of stem-like to effector transition in CAR T cells. Nat. Immunol..

[102] Doan A.E., Mueller K.P., Chen A.Y., Rouin G.T., Chen Y., Daniel B., Lattin J., Markovska M., Mozarsky B., Arias-Umana J. (2024). FOXO1 is a master regulator of memory programming in CAR T cells. Nature.

[103] Li X., Li Y., Dong L., Chang Y., Zhang X., Wang C., Chen M., Bo X., Chen H., Han W., Nie J. (2023). Decitabine priming increases anti-PD-1 antitumor efficacy by promoting CD8+ progenitor exhausted T cell expansion in tumor models. J. Clin. Investig..

[104] Wang Y., Tong C., Dai H., Wu Z., Han X., Guo Y., Chen D., Wei J., Ti D., Liu Z. (2021). Low-dose decitabine priming endows CAR T cells with enhanced and persistent antitumour potential via epigenetic reprogramming. Nat. Commun..

[105] Zhong M., Gao R., Zhao R., Huang Y., Chen C., Li K., Yu X., Nie D., Chen Z., Liu X. (2022). BET bromodomain inhibition rescues PD-1-mediated T-cell exhaustion in acute myeloid leukemia. Cell Death Dis..

